# Neighborhood infrastructure-related risk factors and non-communicable diseases: a systematic meta-review

**DOI:** 10.1186/s12940-022-00955-8

**Published:** 2023-01-05

**Authors:** Yuyang Zhang, Ningrui Liu, Yan Li, Ying Long, Jill Baumgartner, Gary Adamkiewicz, Kavi Bhalla, Judith Rodriguez, Emily Gemmell

**Affiliations:** 1 School of Architecture, Tsinghua University, Beijing, China; 2 School of Architecture and Hang Lung Center for Real Estate, Key Laboratory of Eco Planning & Green Building, Ministry of Education, Tsinghua University, No. 1 Qinghuayuan, Haidian District, Beijing, 100084 China; 3 Institute for Health and Social Policy & Department of Epidemiology, Biostatistics and Occupational Health, McGill University, Montréal, Canada; 4 Department of Environmental Health, Harvard T.H. Chan, School of Public Health, Boston, MA USA; 5 Public Health Sciences, University of Chicago, Chicago, IL USA; 6 Graduate School of Design, Harvard University, Boston, USA; 7 School of Population and Public Health, University of British Columbia, Vancouver, Canada

**Keywords:** Green space, Walkability, Proximity to major roads, Proximity to landfills, Cardiovascular disease, Type II diabetes

## Abstract

**Background:**

With rapid urbanization, the urban environment, especially the neighborhood environment, has received increasing global attention. However, a comprehensive overview of the association between neighborhood risk factors and human health remains unclear due to the large number of neighborhood risk factor–human health outcome pairs.

**Method:**

On the basis of a whole year of panel discussions, we first obtained a list of 5 neighborhood domains, containing 33 uniformly defined neighborhood risk factors. We only focused on neighborhood infrastructure-related risk factors with the potential for spatial interventions through urban design tools**.** Subsequently, following the Preferred Reporting Items for Systematic Reviews and Meta-Analyses (PRISMA) guidelines, a systematic meta-review of 17 infrastructure-related risk factors of the 33 neighborhood risk factors (e.g., green and blue spaces, proximity to major roads, and proximity to landfills) was conducted using four databases, Web of Science, PubMed, OVID, and Cochrane Library, from January 2000 to May 2021, and corresponding evidence for non-communicable diseases (NCDs) was synthesized. The review quality was assessed according to the A MeaSurement Tool to Assess Systematic Reviews (AMSTAR) standard.

**Results:**

Thirty-three moderate-and high-quality reviews were included in the analysis. Thirteen major NCD outcomes were found to be associated with neighborhood infrastructure-related risk factors. Green and blue spaces or walkability had protective effects on human health. In contrast, proximity to major roads, industry, and landfills posed serious threats to human health. Inconsistent results were obtained for four neighborhood risk factors: facilities for physical and leisure activities, accessibility to infrastructure providing unhealthy food, proximity to industry, and proximity to major roads.

**Conclusions:**

This meta-review presents a comprehensive overview of the effects of neighborhood infrastructure-related risk factors on NCDs. Findings on the risk factors with strong evidence can help improve healthy city guidelines and promote urban sustainability. In addition, the unknown or uncertain association between many neighborhood risk factors and certain types of NCDs requires further research.

**Supplementary Information:**

The online version contains supplementary material available at 10.1186/s12940-022-00955-8.

## Introduction

According to the World Bank, in 2019, the global urban population exceeded 55% of the world’s total population, which continues to increase dramatically even today. With such a large proportion of the population living in cities, the impact of the urban environment on human health has become a growing concern [1, 2]. The neighborhood, often characterized by similar social positions, demographics, and housing characteristics, forms the basic geographical component of a city [3, 4]. Moreover, the neighborhood is the most appropriate spatial unit for predicting residents’ daily activities and various exposures to the urban environment, as it is the outdoor space to which they are most frequently exposed [5–7]. Enormous systematic reviews and meta-analyses have examined and confirmed the associations between certain neighborhood risk factors (e.g., green space, walkability, and proximity to major roads) and multiple human health outcomes [8–10].

Although numerous relevant individual studies, systematic reviews, and meta-analyses have been published, studies providing a comprehensive overview of the associations between human health and neighborhood risk factors remain limited. Due to the large numbers of neighborhood risk factor–human health outcome pairs, it is challenging to reveal all health impacts of each neighborhood risk factor. Therefore, in this study, we begin with a subset of neighborhood risk factors that are considered high priority and can be easily modified from the perspective of engineering.

Across various neighborhood environment domains, neighborhood-level socioeconomic status (nSES) is recognized as a major determinant of human health [11]. There is broad consensus that, on average, residents from socially and economically deprived neighborhoods experience worse health outcomes than those from more prosperous areas [12]. Studies have found that residents of high-poverty areas suffer from higher rates of heart disease [13], respiratory ailments [14], cancer [15], and overall mortality [12]. A socially deprived neighborhood is often characterized by poor infrastructure and insufficient medical resources, which may be associated with serious adverse health outcomes for residents [16].

Neighborhood infrastructure refers to the collection of physical facilities that support and sustain the lives and work of people [17], which covers a wide variety of urban physical elements (e.g., parks, roads, and shops). The cyclical decay of many city parks and neighborhoods has rendered some of them unusable and a frequent haven for criminal activity. These issues can only be addressed through changes in government priorities and investments or social mobilization to maintain the infrastructure and preserve the vitality of the space [2]. Globally, infrastructure improvement is an essential issue in urban planning and policy to reduce neighborhood inequity, which requires huge annual investments. Hence, relevant neighborhood infrastructure-related risk factors with strong evidence should be identified, and those with poor conditions should be prioritized for improvement. Assessing the benefits of infrastructure improvement to neighborhood health also supports lobbying for more public investment in infrastructure. Following the place-based interventions conducted a decade ago [18], corresponding spatial interventions for neighborhoods, which can be accomplished using urban design tools, are urgently required to promote sustainable urban development [19].

According to the 2017 World Health Statistics Report issued by the World Health Organization, 71% of total deaths worldwide were caused by chronic non-communicable diseases (NCDs) [20]. Herein, we aim to outline the associations between neighborhood risk factors and human health by starting with neighborhood infrastructure-related risk factors and NCD outcomes.

Regarding the numerous neighborhood infrastructure-related risk factors and NCD outcome pairs, we adopted the meta-review method to comprehensively analyze the evidence on the health impacts of these risk factors. A meta-review (or “review of reviews”) is used to comprehensively assess many neighborhood infrastructure-related risk factors, since there is a high volume of systematic reviews and meta-analyses focused on different neighborhood risk factor–human health outcome pairs [21]. To reduce neighborhood inequity, we addressed the following questions: (1) which neighborhood infrastructure-related risk factors have strong evidence and should be considered high priority for future interventions and (2) which risk factors have inconsistent findings or have rarely been studied that need further research.

## Methods

### Defining neighborhood domains and their risk factors

**Fig. 1:**
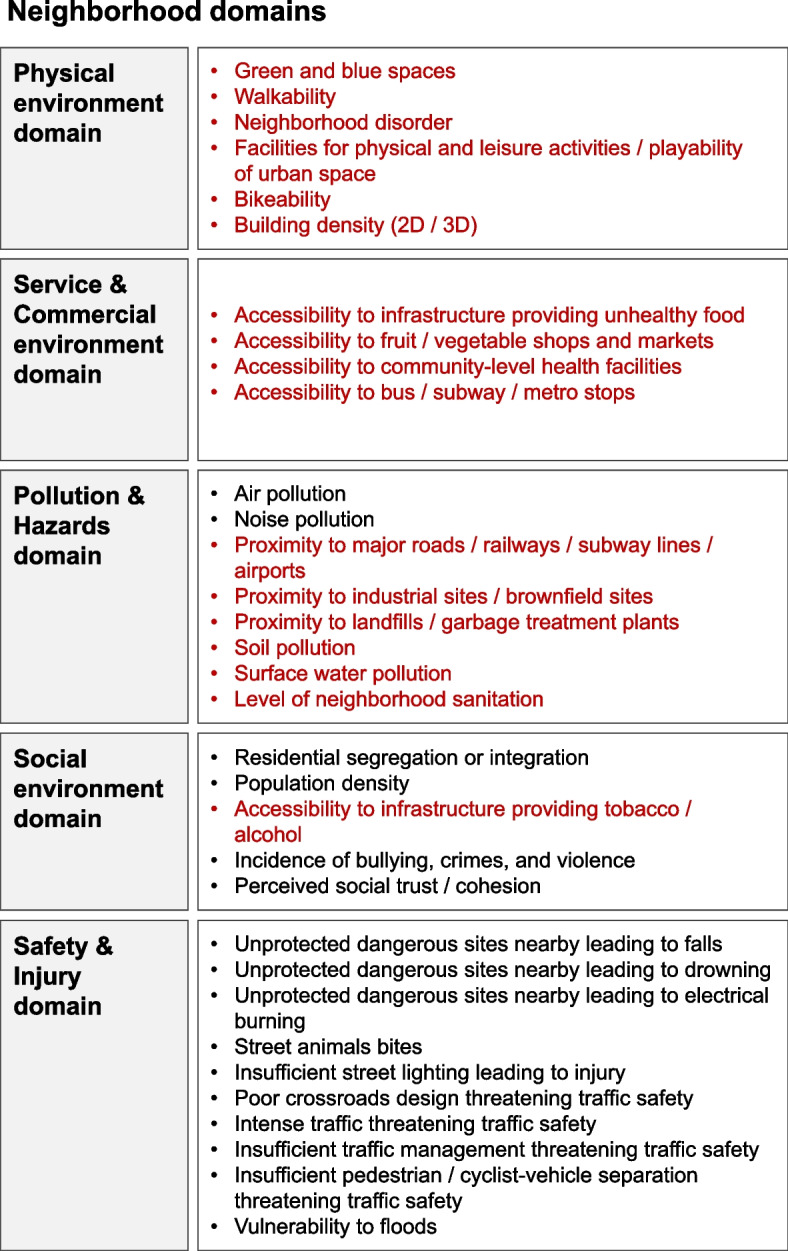
List of neighborhood risk factors, where the risk factors in red are related to infrastructure and are further studied in this meta-review

First, a complete list of neighborhood risk factors was created. This required the researchers to have adequate knowledge of the neighborhood environment, human health, and the pathways bridging the two fields. Therefore, through the *Pathways to Equitable and Healthy Cities* partnership, international workshops were held with experts from Asia, Europe, North America, and Africa, as well as from multidisciplinary backgrounds, including public health and urban science. Referring to domain identification in [22], to identify potential risk factors, a scoping review was conducted on the National Center for Biotechnology Information (NCBI) web database PubMed using the keywords “neighborhood environment” and “health outcome.” Then, based on the review and professional knowledge, the participants conducted several rounds of discussions through online meetings and emails from July 2019 to June 2020. Finally, a list of 5 neighborhood environment domains, which contained 33 uniformly defined neighborhood or area risk factors, was created. This list is shown in Fig. 1, and more detailed information is presented in Additional file 1: Table S1. The five neighborhood environment domains are listed as follows: physical environment, service and commercial environment, pollution and hazards, social environment, and safety and injury.

In this study, we only focused on the neighborhood infrastructure-related risk factors with the potential for spatial interventions through urban design tools, which indicates that the states of these risk factors (including shape, layout, density, and scale) can be modified through design and engineering to create better residential neighborhoods [23]. The risk factors in the safety and injury domain were not considered because we only focused on chronic NCDs. Finally, 17 risk factors were included in this meta-review: green and blue spaces, walkability, neighborhood disorder, facilities for physical and leisure activities/playability of urban space, bikeability, building density, accessibility to infrastructure providing unhealthy food, accessibility to infrastructure providing fruit/vegetable shops and markets, accessibility to community-level health facilities, accessibility to bus/subway/metro stops, proximity to major roads/railways/subway lines/airports, proximity to industry/brownfield sites, proximity to landfills/garbage treatment plants, soil pollution, surface water pollution, level of neighborhood sanitation, and accessibility to infrastructure providing tobacco and alcohol. These risk factors are marked in red in Fig. 1.

### Search strategy

**Fig. 2:**
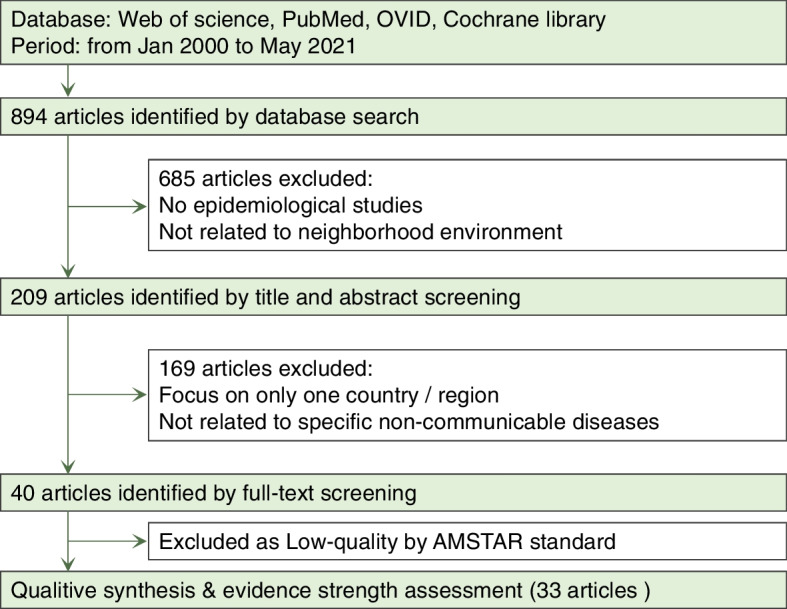
PRISMA flowchart of the literature search and screening

We searched the following four databases for articles from January 2000 to May 2021 on June 15, 2021: Web of Science, PubMed, OVID, and Cochrane Library. Only systematic reviews and meta-analyses were included in this meta-review. The search keywords were (“systematic review” OR “meta-analysis”) AND (“health” OR “disease” OR “obesity” OR “birth”) AND (KEYWORDS for each risk factor); the keywords for each risk factor are listed in Additional file 1: Table S2. The language was limited to English, and only published studies were included. All studies identified through the database search were screened according to their titles and abstracts. A study was excluded if it was (1) not an epidemiological study or (2) not related to the neighborhood environment. Then, through a full-text screening of the remaining studies, the following were excluded: (1) one country-specific or one region-specific study (for general principle) or (2) not related to specific NCDs or all-cause mortality, such as mental health, obesity, birth-related outcomes, physical activity, and self-reported general health. Finally, the selected studies were included in this meta-review and a later quantitative synthesis. The flowchart describing this process is presented in Fig. 2. This meta-review was conducted following the Preferred Reporting Items for Systematic Reviews and Meta-Analyses (PRISMA) guidelines (www.prisma-statement.org).

### Quality assessment of included reviews

After the screening, a quality assessment was conducted for all included reviews according to A MeaSurement Tool to Assess Systematic Reviews (AMSTAR) [24]. AMSTAR provides a checklist of 11 questions to evaluate the quality of each systematic review and meta-analysis. The AMSTAR checklist is provided in Additional file 1: Table S3. If the answer to a question was “Yes,” the review was given an additional score; otherwise, it was given a zero score. Finally, the total score of each included review was calculated, and according to the scores, all included reviews were classified as very high quality, high quality, moderate quality, or low quality. The classification criteria are listed in Table 1. As questions 9 and 10 in the checklist were only applicable to meta-analysis, the classification criteria for the meta-analysis were slightly different from those of the systematic review. If a review received a quality score less than or equal to 5 for the meta-analysis and 4 for the systematic review, it was classified as low quality and excluded from the data extraction and qualitative synthesis later [25]. Although these cut-offs are relatively casual, they play a qualitative role in facilitating the structure of the literature [26]. Low-quality reviews were excluded because the low scores resulted from their lack of following a protocol or failure in considering the risk of bias [27]; thus, these results and conclusions need to be interpreted with caution. The quality assessment was independently conducted by two reviewers. If different scores were assigned to the same review by the two reviewers, the disagreement was resolved through discussion. The quality assessment results are shown in Additional file 1: Table S4.

**Table 1:** Classification criteria of quality assessment for the included reviews

**Quality**	**Meta-analysis (score)**	**Systematic review (score)**	**Outcome**
**Very high**	10–11	8–9	Included for data extraction later
**High**	8–9	7	
**Moderate**	6–7	5–6	
**Low**	1–5	1–4	Excluded

### Data extraction and evidence evaluation

The following characteristics were extracted from moderate-quality and high-quality systematic reviews and meta-analyses: population characteristics, specific NCDs, neighborhood risk factors examined, range of sample sizes, number of studies of each study type, main findings, and findings for the subgroup population. The data for the primary studies included in each review were extracted from the reviews, not from the primary studies themselves. Certain reviews reported a range of risk factors, some of which were not relevant to our focus. In this case, we separated data related only to the specific factors we were concerned about. This step excluded the same studies that were included in different reviews and addressed the main drawback of the meta-reviews [28]. All data were extracted by one reviewer and checked by another reviewer. When different reviews reported the same study, we prioritized the findings in the high-quality review.

For each neighborhood risk factor–health outcome pair in the included reviews, the evidence was evaluated through two steps. First, the main findings were summarized and graded into four types: “harmful,” “protective,” “null,” and “inconsistent” [29]. “Null” indicated that no associations were observed between this neighborhood risk factor and the NCD outcome. “Inconsistent” indicated that the individual studies in this systematic review or meta-analysis did not offer consistent conclusions about the risk factor–outcome relationship. The grading of “harmful,” “protective,” “null,” and “inconsistent” was based on the conclusions in the systematic reviews or meta-analyses instead of being determined by the two reviewers.

Second, since the quality of the review differed from that of the evidence, we also evaluated the strength of the evidence. The evidence was categorized into four levels: strong, medium, weak, and limited [25]. “Limited” represented that the evidence was supported by only one study in a review. The other strength types were evaluated by weighting the study scores for different study types, as shown in Table 2. In detail, a cohort study was given 3 points, a case–control study or case-crossover study was given 2 points, an ecological or a time-series study was given 1 point, and a cross-sectional study or survey was given 0 points [30]. A cut-off score of 9 for strong evidence implied evidence obtained from at least 3 cohort studies or 2 cohort studies and 2 case–control studies, indicating high confidence in the evidence. A cut-off score of 2 for weak evidence implied evidence obtained from 1 case–control study or 2 ecological studies or several cross-sectional studies, but no cohort study, indicating low confidence in the evidence. The other scores of 3–8 for medium evidence indicated that the evidence was from 1–2 cohort studies or 2–4 case–control studies, indicating moderate confidence in the evidence. For example, one meta-analysis regarded green space as a protective factor against cardiovascular disease (CVD), supported by seven individual studies, including two cohort studies, four ecological studies, and one cross-sectional study. This evidence strength was evaluated as “strong” in our study, since the average score was 2x3+4x1+1x0 = 10.

**Table 2:** Criteria for evidence strength

**Strength**	**No. of supporting individual studies**	**Average score, *S***
**Limited**	≤ 1	
**Weak**	≥ 2	0 ≤ *S* ≤ 2
**Medium**	≥ 2	2 < *S* < 9
**Strong**	≥ 2	9 ≤ *S*

## Results

As shown in Fig. 2**,** the systematic search identified 894 different systematic reviews, of which 685 were judged as not meeting the inclusion criteria based on their abstracts. Of the remaining 209 potentially eligible systematic reviews, 169 were further excluded, as they did not meet the inclusion criteria when full-text versions were examined. Finally, through the quality assessment, 7 reviews were further excluded and 33 reviews were left (Additional file 1: Table S4), among which 13 were rated as moderate quality, 18 as high quality, and 2 as very high quality. Altogether, these reviews analyzed 481 individual studies.

Fourteen of the 33 systematic reviews focused on the associations between NCDs and proximity to major roads [10, 31–43], 11 on green space [44–54], 4 on walkability [41, 43, 44, 51], 4 on accessibility to infrastructure providing unhealthy food [41, 43, 51, 55], 6 on proximity to industry [56–61], 3 on facilities for physical activity or recreation [43, 44, 51], and 2 on proximity to landfills [57, 62]. Most reviews included studies that measured multiple neighborhood risk factors and NCD outcomes (Additional file 1: Table S5). The results obtained for the subgroups were also extracted and are demonstrated in Additional file 1: Table S6.

### Findings from high-quality reviews

Of the 33 reviews included, 20 were classified as high quality and very high quality. Among these, 10 reviews focused on green and blue spaces [44–49, 51, 52], 5 on proximity to major roads or high-traffic roads [10, 35, 40, 41, 63], 5 on proximity to industry [56, 58–61], 3 on walkability [41, 44, 51], and 2 reviews each focused on access to infrastructure providing unhealthy food [41, 51] and facilities for physical and leisure activities [44, 51]. There was only one high-quality review on proximity to landfills [57]. Most high-quality systematic reviews and meta-analyses provided medium or strong evidence. The pieces of evidence and their strengths are shown in Table 3. Overall, 54 risk factor–outcome pairs of all evidence strength levels were identified from high-quality reviews.

**Table 3:** Grading level and evidence strength of very high-and high-quality reviews

**Risk factors**	**NCD outcomes**			
	**Harmful**	**Protective**	**Null**	**Inconsistent**
**Green and blue spaces**		Kidney disease (-) Prostate cancer (-) Lung cancer ( +) Cancer (-) Asthma ( +) **Atopic diseases (+ + +)** **Respiratory diseases (+ + + , + + + , + + +)** **T2DM** (+ + , + + , + ** + + , + + +**) **Stroke (+ + + , + + +)** **CHD (+ +)** **IHD (+ + +)** **CVD** (-, -, + ** + + , + + + , + + + , + + +**)		T2DM (+ +)
**Walkability**		CHD (-) **T2DM** (+ , + ** + + , + + +**)	CHD (-)	
**Facilities for physical and leisure activities**		**T2DM** (+ +)	**CHD (+ + +)** **Stroke (+ + +)**	**T2DM (+ + +)**
**Accessibility to infrastructure providing unhealthy food**	Stroke (+ +) CVD (+ +)			**T2DM (+ + +)**
**Proximity to major roads**	CHD (-) CVD (-, + +) Rheumatoid arthritis (RA) (+ +) **Childhood leukemia (+ + + , + + +)**			
**Proximity to industry**	Respiratory tract diseases (+ +) **Lung cancer (+ + +)** **Leukemia (+ + + , + + +)**			CVD (+ +) **Non-Hodgkin’s lymphoma (NHL) (+ + +), Hodgkin’s lymphoma (HL) (+ + +), multiple myeloma (MM) (+ + +)**
**Proximity to landfills**	Asthma (+ +) Breast cancer (+ +) **Liver cancer (+ + +)** **Bladder cancer (+ + +)** **NHL (+ + +)**			

*Note*: In the brackets, +  +  + means “strong.” +  + means “medium,” + means “weak,” and—means “limited.” The NCDs in bold show strong evidence

#### Green and blue spaces

Nine high-quality reviews examined the associations between NCDs and green and blue spaces, which accounted for half of all high-quality reviews. The term green space refers to vegetation (e.g., trees, grass, forests, and parks), whereas blue space refers to all visible surface waters in space (e.g., lakes, rivers, and coastal water). Thirteen high-quality reviews identified strong evidence of the protective effects of green and blue spaces on multiple NCD outcomes, including atopic diseases, respiratory diseases, T2DM, stroke, coronary heart disease (CHD)/ischemic heart disease (IHD), and CVD. For example, Yuan et al. [48] conducted a meta-analysis and found a statistically significant reduction for stroke mortality [pooled HR (95% CI) = 0.77 (0.59, 1.00)] but no significant reduction for CVD mortality [pooled HR (95% CI) = 0.99 (0.89, 1.09)], IHD mortality [pooled HR (95% CI) = 0.96 (0.88, 1.05)], and respiratory disease mortality [pooled HR (95% CI) = 0.99 (0.89, 1.10)] for a 0.1 unit increase in the normalized difference vegetation index (NDVI) around residences. The NDVI quantifies vegetation by measuring the difference between near-infrared light (which is strongly reflected by vegetation) and red light (which is adsorbed by vegetation). Another meta-analysis [49] reported a statistically significant decrease in stroke incidence and T2DM. In a review of urban green space and human health [46], the risks of CVD and respiratory mortality were negatively associated with green space, although there were not many studies examining the associations.

Medium evidence was found for CHD and T2DM in four high-quality reviews. A meta-analysis conducted by Twohig-Bennett et al. [49] reported a statistically significant reduction in type II diabetes (OR = 0.72; 95% CI, 0.61, 0.85) and a reduction in CHD incidence (OR = 0.92; 95% CI, 0.78–1.07). For lung cancer, green space had a protective effect, but the evidence was weak, which included two ecological studies and one cross-sectional study [45]. Evidence of the protective effect of green space on kidney disease and prostate cancer was limited, and only one individual study was found for each [46].

#### Walkability

Four high-quality reviews examined the effects of neighborhood walkability on T2DM. Strong evidence was reported in a meta-analysis [44] that included three cohort studies examining the association between walkability and T2DM. The same protective effects were found in another complex meta-analysis that assessed the association between NCDs and the neighborhood built environment [44]. The protective effect of walkability on T2DM was confirmed across six longitudinal studies. Neighborhood walkability was negatively associated with the risk of T2DM (OR = 0.79; 95% CI, 0.7–0.9). In addition, two pieces of limited evidence were reported in the same review [44], which found no association between street connectivity and CHD but found higher land use mix as a protective factor for CHD.

#### Facilities for physical and leisure activities

Two reviews examined the association between NCDs and facilities for physical and leisure activities. One of them focused on recreational facilities, while the other focused on facilities for physical activities. Chandrabose et al. [44] showed strong evidence that access to recreational facilities had no effect on CHD in three cohort studies and a protective effect on diabetes outcomes in two cohort studies. However, this review claimed that there were insufficient studies to draw a clear conclusion, which in our study was rated as medium-strength evidence. den Braver et al. [51] found an inconsistent association between T2DM and the facilities for physical activities in three longitudinal studies and three cross-sectional studies, which was rated as strong evidence. Two of the six studies indicated that more neighborhood resources available for physical activities were associated with a lower risk of T2DM, while the other four did not observe any association between physical activities and T2DM.

#### Accessibility to infrastructure providing unhealthy food

Two high-quality systematic reviews explored the relationship between NCDs and an unhealthy food environment. den Braver et al. [51] found no association between diabetes and an unhealthy food environment after reviewing 7 longitudinal studies and 13 cross-sectional studies, which was evaluated as strong evidence. Malambo et al. [41] identified the harmful effect of high fast-food restaurant availability on stroke and CVD from one longitudinal study and one cross-sectional study among Mexican–American adults, but the harmful effect was not observed among non-Hispanic White adults. Both pieces of evidence were evaluated as having medium strength.

#### Proximity to major roads

Five high-quality systematic reviews or meta-analyses examined the associations between NCDs and proximity to major roads (heavy traffic).

A meta-analysis [32] identified 26 studies, in which 19 case–control studies and 1 cohort study focused on residential traffic exposure; this was considered strong evidence. The meta-analysis found that residential exposure to heavy traffic roads could lead to childhood leukemia but only in the highest exposure category. Boothe et al. [10] identified the same result across nine studies, including eight case–control studies and one population-based study, and reported that childhood leukemia was associated with residential exposure to high traffic density during the postnatal period. Moreover, the harmful effects did not differ by study location, study period, type of exposure metric, cancer type, control for SES, or quality score.

A meta-analysis [35] that rheumatoid arthritis (RA) was associated with residential exposure to heavy traffic during the postnatal period [pooled relative risk (RR) = 1.34, 95% CI: 1.11–1.62], which was identified through two studies, a prospective cohort study and a nested case–control study; this was considered medium-strength evidence. Another medium-strength evidence was reported by a systematic review of eight cross-sectional studies and two cohort studies that found harmful effects of proximity to major roads for CVD [40]. Malambo et al. [41] conducted a systematic review of the effects of complex neighborhood environment characteristics on major CVD outcomes. In this review, two limited pieces of evidence of harmful effects were found for CVD and CHD.

#### Proximity to industry

Six high-quality reviews examined the effects of proximity to industry. Two very high-quality meta-analyses reported increased risks of both mortality and morbidity of leukemia among residents living near petrochemical industrial complexes (PICs), which indicates strong evidence. Boonhat and Lin [56] found that higher RRs of leukemia incidence existed with follow-up periods of ≥ 10 years. In addition, Jephcote et al. [60] reported inconsistent findings for three NCD outcomes: Hodgkin’s lymphoma (HL) (RR = 1.03, 95% CI = 0.81–1.30), non-Hodgkin’s lymphoma (NHL) (RR = 1.06, 95% CI = 0.97–1.17), and multiple myeloma (MM) (RR = 1.16, 95% CI = 0.83–1.63). Lin et al. [61] conducted a meta-analysis of residential proximity to PICs for lung cancer across six cohort studies and one case–control study, which indicated a slightly higher risk of lung cancer mortality (RR = 1.03, 95% CI = 0.98–1.09). Lin et al. [58] conducted another meta-analysis of residential proximity to PICs for lung cancer across six cohort studies. The results showed a 19% higher risk of lung cancer for residents living close to PICs (95% CI = 1.06–1.32). The subgroup analysis was conducted by gender and location. Higher risks were found for females and groups in Europe. In addition, two pieces of medium-strength evidence were identified by Raffetti et al. [59], who found an increased risk of respiratory tract diseases, as well as an inconsistent effect on CVD, for residents living close to the plant.

#### Proximity to landfills

Only one systematic review [62] that examined the association between NCDs and proximity to landfills was assessed as a high-quality review. Three strong-and two medium-strength evidence were identified. For strong evidence, liver cancer, bladder cancer, and NHL were reported to be positively associated with living close to landfills. For medium evidence, the review reported a harmful effect for asthma and breast cancer.

### Findings from moderate-quality reviews

Of the 33 reviews included, 13 were classified as moderate quality. Among these, three reviews focused on green and blue spaces [43, 50, 54], nine on proximity to major roads or high-traffic roads [32–34, 36–39, 42, 43], and two on accessibility to infrastructure providing unhealthy food [43, 55]. Proximity to landfills [62], walkability [43], and facilities for physical and leisure activities [43] were studied by one moderate-quality review each. Overall, most moderate-quality systematic reviews and meta-analyses provided strong or medium evidence. The grading level and evidence strength of moderate-quality reviews are shown in Table 4. Overall, 26 risk factor–outcome pairs at all evidence strength levels were identified from moderate-quality reviews.

**Table 4:** Grading level and evidence strength of moderate-quality reviews

**Risk factor**	**NCD outcomes**			
	**Harmful**	**Protective**	**Null**	**Inconsistent**
**Green and blue spaces**		T2DM (+ + , + +) Cancer ( +)	T2DM ( +)	**Atopic diseases (+ + +)** CVD (+ +)
**Walkability**		**T2DM (+ + +)**		
**Facilities for physical and leisure activities**			**T2DM (+ + +)**	
**Accessibility to infrastructure providing unhealthy food**	Stroke (-)			**T2DM (+ + +)**
**Proximity to major roads**	CVD (-) Asthma (-, -) RA (+ +) **Lung cancer** (+ + +) **T2DM** (-, + + , + + +) **Leukemia** (+ + , + ** + +**) Dementia (-, + +)			**Dementia (+ + +)** T2DM (+ +)
**Proximity to landfills**	CVD (+ +) Respiratory diseases (+ +)			

In the brackets, +  +  + means “strong,” +  + means “medium,” + means “weak,” and—means “limited”

### Green and blue spaces

Three moderate-quality systematic reviews included seven neighborhood infrastructure-related risk factors and specific disease pairs, discussing the associations among green and blue spaces and CVD, T2DM, atopic diseases, and cancer. There was only one strong evidence identified from the review [50], which concluded that green space was an inconsistent risk factor, as greenness significantly improved the health status for atopic diseases (asthma, eczema, and rhinitis) in only 4% of the available studies. The same review [50] also reported other medium-strength evidence that exposure to greenness significantly decreased the risk of diabetes in 58% of individual studies in their systematic reviews, suggesting that green space is a protective factor for diabetes. However, the review [50] reported an inconsistent effect of green space on CVD, as only 18% of studies found a reduction effect. Other medium-strength evidence was examined for associations with T2DM. Dendup et al. [43] found an overall protective effect of green space/open space on diabetes. All three cross-sectional studies showed that the incidence of diabetes among residents in greener neighborhoods was significantly reduced, and the other three studies also showed the protective effect of greenness, although not significant.

Browning et al. [50] concluded that greenness significantly decreased the risk of T2DM in 58% and CVD in 18% studies; only the latter evidence had an inconsistent conclusion. The systematic review of Gascon et al. [54] was the only one concerning exposure to blue space. Nevertheless, no significant associations between diabetes and proximity to blue space were observed in the two studies that investigated this association [RR_1_ = 1.86 (0.69, 1.06), RR_2_ = 0.88 (0.65, 1.20)].

### Walkability

There was only one moderate-quality systematic review concerning walkability. Dendup et al. [43] summarized four cohort studies, one ecological study, and two cross-sectional studies and concluded that a higher level of walkability was associated with a lower risk of T2DM. These authors considered walkability to be a protective factor, and the evidence strength was medium. This conclusion was not consistent with those drawn from high-quality reviews.

### Facilities for physical and leisure activities

Only one moderate-quality systematic review focused on the association between T2DM and access to physical activity facilities [43]. Dendup et al. [43] reported a cohort study, which combined the method of Geographic Information System (GIS) and surveys, and observed a significant reduction of 19% in the risk of T2DM for an interquartile increase in physical activity resources, while six other related studies found no significant association between diabetes and availability/distance to physical activity resources. Therefore, this review was graded as null, and the evidence was evaluated as strong. However, two high-quality systematic reviews reported facilities for physical and recreational activities as a protective factor and an “inconsistent” factor, respectively. The large divergence of these reviews indicates that more studies are required to better understand the relationship between T2DM and access to physical/recreational activity resources.

### Accessibility to infrastructure providing unhealthy food

There were two systematic reviews on accessibility to infrastructure providing unhealthy food. Dendup et al. [43] provided strong evidence that the available individual studies showed an inconsistent association between T2DM and unhealthy food access. Six studies found that more access to healthy food was beneficial in lowering the risk of T2DM, while ten other studies did not find any significant association. The “inconsistent” conclusion from this moderate-quality review agreed with that obtained from the high-quality review. In addition, Kraft et al. [55] conducted a systematic review on the influence of the neighborhood unhealthy food environment on the health of low-SES populations in the United States. They found that unhealthy food access is significantly positively associated with the risk of stroke for Mexican–American adults, which was rated as medium-strength evidence. Current epidemiological studies on unhealthy food environments have focused more on obesity outcomes, neglecting the influence on NCDs.

### Proximity to major roads

The number of reviews for proximity to a major road ranked top among all moderate-quality reviews. There were four pieces of strong evidence, four pieces of medium-strength evidence, and 5 pieces of limited evidence. For strong evidence, Zhao et al. found that an increased risk of T2DM was observed for residents living near major roadways. The meta-analysis suggested that the adjusted pooled RR for residential proximity to major roadways was 1.12 (95% CI: 1.03–1.22). Hamra et al. [39] found that distance to roadways had an increased risk of lung cancer, which may be due to exposure to high level of air pollution.

For medium-strength evidence, Dzhambov et al. [36] synthesized one prospective cohort study and one nested case–control study and found that a higher risk of RA was observed for people living within 50 m of a heavy traffic road. The adverse effect of proximity to major roads on RA was consistent with that reported by high-quality systematic reviews. In addition, Peters et al. [34] reported that the association between dementia and proximity to major roads was inconsistent, among which one study found a negative effect of proximity to major roads, while another study obtained insignificant results. Filippini et al. [32] discovered that, for childhood leukemia, the pooled odds ratio of exposure to residential traffic density and proximity to petrol stations/repair garages was 1.07 (95% CI: 0.93–1.24) and 1.83 (95% CI: 1.42–2.36), respectively. This implies that proximity to a major road is a harmful factor for childhood leukemia. Dendup et al. [43] stated that the association between diabetes and distance to roadways was inconsistent because three studies showed a significantly harmful effect, while others showed no significant difference or no difference in the risk of T2DM.

For limited evidence, Delgado-Saborit et al. found that residential traffic exposure increased the risk of dementia. Both Gasana et al. [37] and Salgado et al. [38] indicated that proximity to major roads increased the risk of asthma in children and adults. Salgado et al. [38] regarded road density in the neighborhood as a harmful factor for CVD mortality. High traffic intensity in the neighborhood also increased the risk of type II diabetes. The limited evidence provided us with a rough picture of the health effects of these risk factors; thus, higher-quality systematic reviews or meta-analyses are required to validate the conclusions.

### Proximity to landfills

One systematic review focused on the association between residential exposure to municipal solid waste and two NCDs (CVD and respiratory diseases). Vinti et al. [62] provided two pieces of medium-strength evidence that residents living near landfills had a higher risk of developing CVD and respiratory diseases. However, this systematic review indicated that most study types were cross-sectional, and there was a lack of cohort studies. In addition, there were no high-quality reviews concerning proximity to landfills; thus, more relevant studies are required to clarify the harmful effects of landfills on urban residents’ health.

## Discussion

The rapid but unbalanced development of the neighborhood environment and its association with residents’ health have recently become important issues. This meta-review comprehensively assessed a wide range of neighborhood infrastructure-related risk factors according to their effects on NCDs. Our synthetic evaluation of the health effects of several neighborhood infrastructure-related risk factors fills this literature gap and can guide relevant spatial interventions to reduce the risk of NCDs.

### Summary of evidence

Table 5 shows the final synthetic evaluation results obtained for the seven neighborhood risk factors presented in the Results section. The synthetic evaluation considered only medium-strength and strong evidence from all the included reviews, as summarized in Additional file 1: Table S5. Given that different reviews may have different conclusions about the same risk factor–outcome pairs because they may include different individual studies, we first present our synthesis principles. When all pieces of evidence for the health effects were in the same direction, we only reported the number of strong evidence pieces in the table, and if there was no strong evidence, we reported the number of medium evidence pieces. This is because strong evidence implies a high level of confidence, whereas medium-strength evidence implies fewer individual studies with a high level of confidence; thus, these results should be interpreted with caution. When the same risk factor–outcome pairs appeared in different directions of health effects, we prioritized the direction of strong evidence and used the direction with most pieces of strong evidence as our final direction. The effect was considered inconsistent when there was an equal number of strong and medium strength evidence pieces with different directions.

**Table 5:**
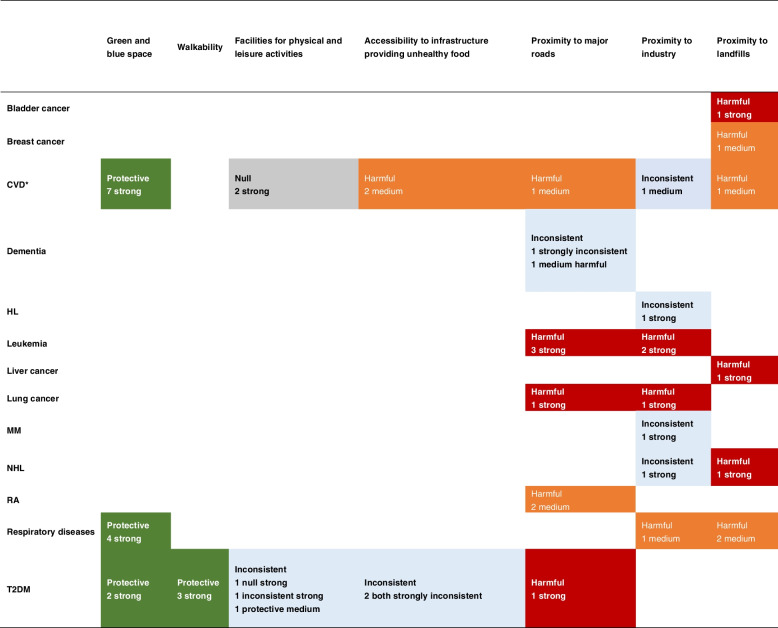
Summary of strong and medium evidence from the included reviews

If the associations between the risk factor and health outcome were consistent in each related review, the corresponding blank in Table 5 was filled with “protective” in green, “harmful” in red and orange, or “null” in grey, according to the specific associations. In contrast, if the associations were inconsistent in one or all reviews, the corresponding blank in Table 5 was filled with “inconsistent” in blue

The number of supporting reviews and their evidence strengths are marked in the blanks

^*^CHD/IHD and stroke were merged into CVD

Seven main NCD outcomes were found to be associated with neighborhood infrastructure-related risk factors. A total of 19 pairs were confirmed to have a definitive health effect, and 1 pair was confirmed to have no association. As shown in Table 5, green and blue spaces or walkability had protective effects on human physical health. In particular, a higher density of green and blue spaces can decrease the risks of CVD, T2DM, and respiratory diseases. A neighborhood with a higher walkability environment can effectively reduce the risk of T2DM. The health benefits of green and blue spaces and high walkability have been discussed in many reviews and individual studies. The role of urban green and blue spaces, such as parks, forests, green roofs, streams, and community gardens, in the provision of regulating services and related health benefits includes urban heat regulation, noise reduction, air quality improvement, moderation of climate extremes, runoff mitigation, waste treatment, pollination, pest regulation, seed dispersal, and global climate regulation [48, 64], which are commonly regarded as protective factors of CVD and other outcomes. Green space and favorable walking built environments also promote physical activities, social interactions, and psychological well-being, thus benefiting the general health of urban residents [49].

In contrast, proximity to major roads, industry, and landfills can pose serious threats to human health. Proximity to major roads has harmful effects on CVD, RA, leukemia, lung cancer, and T2DM. Leukemia and lung cancer can also be induced by long-term exposure to industrial sites. In addition, residential proximity to landfills is associated with CVD, respiratory diseases, breast cancer, lung cancer, liver cancer, bladder cancer, and NHL. The pathways may be related to ambient air toxins emitted from industrial sites (e.g., 1,3-butadiene, benzene, and aromatic hydrocarbons) and major roads (e.g., PM_2.5_, NO_2_/NO_x_, and O_3_), which are mutagenic, exhibit carcinogenic properties, and increase the risk of diseases, thereby affecting human health [34, 56, 60]. The release of hazardous chemicals through leachates from landfills, such as organic chlorinated compounds, heavy metals, and petrochemicals, also has grievous consequences for the surrounding environment and human life [57, 61, 65].

In addition, fast-food stores, which provide high-calorie unhealthy food, are usually considered a risk factor and are identified as harmful for CVD in our study. However, facilities for physical and leisure activities are considered irrelevant to the risk of CVD and are rated as “null.”

### Implications for future studies

Our meta-review confirmed that some neighborhood infrastructure risk factors have health effects on NCD outcomes, with strong evidence of harmful or protective effects. To better understand the risk factor–outcome associations, further research is needed. The main focus is on inconsistent health effects and the medium, weak, and limited evidence, for each of which we have a corresponding strategy.

For risk factor–outcome pairs of inconsistent health effects, seven pairs were identified, including facilities for physical and leisure activities and T2DM, accessibility to infrastructure providing unhealthy food and T2DM, proximity to major roads and dementia, and proximity to industry and CVD, HL, MM, and NHL. All these inconsistent conclusions summarized from the included reviews are attributed to the fact that most of the studies did not observe an association, and there were no conflicting conclusions. These inconsistent results suggest that more evidence is needed to understand the associations between the specific risk factors and these NCD outcomes. For facilities for physical and leisure activities and T2DM pair, all three health effects, i.e., null, protective and inconsistent, were found; thus, further exploration is needed.

For medium-strength evidence, seven pairs were identified, including accessibility to infrastructure providing unhealthy food and CVD, proximity to major roads and CVD and RA, proximity to industry and respiratory diseases, and proximity to landfills and breast cancer, CVD, and respiratory diseases. For these pairs, some individual studies already exist, and the directions of health effects are consistent. However, there is a lack of reliable studies, so the confidence interval of these risk factor–outcome pairs should be improved with more cohort or case–control studies.

For the weak and limited evidence (i.e., only one individual study or only a cross-sectional study type), there were three pairs, all of which were protective: green and blue spaces and kidney disease, prostate cancer, walkability, and CVD. For these pairs, even at the beginning, there were some promising results, especially for the walkability and CVD pair, as CVD was confirmed to be associated with physical activity and expected to be strongly associated with the neighborhood walking environment.

Other types of infrastructure have not appeared in any reviews, including some common infrastructure types that can affect physical activities and human health (e.g., subway lines and stations, bike lanes, and infrastructure that provides tobacco and alcohol), suggesting that a systematic review and meta-analysis of related topics could be conducted next.

### Implications for infrastructure policy

Clarifying the hazards and health effects of infrastructure configuration can provide support for government policy priorities and the health effects of neighborhoods can result in more infrastructure investment. The findings of this review provide a reference for this purpose. For example, in terms of factors with harmful effects, new constructions of hazardous and polluted industries and waste landfills should be restricted in densely populated urban areas. For those already established, a wide green belt planted with tall trees should be built in the surroundings to reduce air pollution. Meanwhile, the highly polluted industries and large waste landfills in densely populated urban areas should be immediately moved to peri-urban areas. For newly built major roads, the distance from buildings alongside the road should be sufficient to plant a wide green belt. For existing major roads, more and higher road-adjacent trees should be planted to reduce traffic-related air pollution, noise, and the heat island effect. In terms of factors with protective effects, green infrastructure has numerous benefits not only for human health but also for improving the urban environment. Although it can mitigate the harmful effects of some urban infrastructure, parks and other green infrastructure for promoting physical activities should be restricted near the infrastructure with harmful effects, including polluted industry, landfills, and major roads, to reduce exposure to the general population. In addition to increasing the urban green space coverage, green morphology variables, such as the shape and aggregation index, should also be considered.

### Strengths and limitations

The rapid but unbalanced development of neighborhood environments and their associations with residents’ health have recently become important public issues. This study comprehensively assessed a wide range of neighborhood infrastructure-related risk factors according to their effects on NCDs. The synthesis of reviews in this study provides a summary of available evidence and identifies key neighborhood infrastructure-related factors from the perspective of public health.

The first strength of this study is that we provided a complete list of neighborhood risk factors with clear explanations that are not limited to infrastructure-related risk factors and can be used as a reference for future studies. Second, we analyzed each original study in each included systematic review in detail, which is not usually required in a meta-review, in order to precisely evaluate the evidence strength of these neighborhood risk factors. This helped us eliminate the influence of duplicate studies in multiple systematic reviews and to accurately extract and categorize the associations between different risk factors and health outcomes when some systematic reviews covered multiple risk factors and outcomes. Third, we distinguished the quality of evidence from the quality of systematic reviews. Previous meta-reviews have focused more on the quality of included reviews, while the strength of the evidence provided on various health associations from these reviews can be quite different from the quality of reviews. Therefore, in the final synthesis results obtained in this study, evidence with weak strength or a limited number of studies was excluded to enhance the robustness and reliability of the conclusions.

However, this study has several limitations. This meta-review did not provide a systematic search of all individual studies on the associations between NCDs and neighborhood infrastructure-related risk factors. Considering only systematic reviews and meta-analyses may limit the focus on neighborhood risk factors that have been studied the most while neglecting the health impacts of risk factors that only have individual studies and no related systematic reviews. Future studies should focus more on other neighborhood risk factors and conduct related systematic reviews and meta-analyses. Additionally, the large number of positive findings reported may result from possible publication bias; therefore, the results need to be interpreted with caution. However, it is currently difficult to evaluate the publication bias because most systematic reviews related to neighborhood risk factors are narrative systematic reviews instead of quantitative synthesis, such as meta-analysis. More quantitative systematic reviews are required in the field of healthy neighborhoods and cities. Furthermore, the definitions and terms of neighborhood risk factors were so diverse and inconsistent that the search terms applied in this study might have missed some related reviews. Finally, our approach to evaluating evidence strength did not afford much consideration to the specific study design of each individual study because we extracted information from the reviews rather than the original individual studies, which places high requirements on the approach to evaluating evidence strength in reviews. Here, in response to the integration of evidence from observational studies, we propose that future reviews carefully consider the informative study design of each risk factor–outcome pair to better synthesize the evidence [66–68].

## Conclusions

This meta-review was intended to present a comprehensive overview of neighborhood infrastructure-related risk factors on NCDs, which seems to be impossible to achieve in one systematic review or meta-analysis. Overall, the neighborhood is the outdoor space to which humans are most frequently exposed, and thus, is a crucial determinant of human health. Findings on neighborhood infrastructure-related risk factors with strong evidence can help improve healthy city guidelines and promote urban sustainability. Additionally, the associations between many neighborhood risk factors and certain types of NCDs remain unknown or uncertain, which is in urgent need for further research.

## Supplementary Information

**Additional file 1: Table S1.** Explanation, measurement method and potential data source for neighborhood risk factors. **Table S2.** The search keywords for each neighborhood risk factor. **Table S3.** The checklist of AMSTAR. **Table S4.** Quality assessment results of all the included reviews. **Table S5.** Characteristics of all the included reviews. **Table S6.** Summary of reviews that compare findings for different population subgroups.

## Data Availability

Not applicable.
