# Supplementary material for: Neighborhood infrastructure-related risk factors and non-communicable diseases: a systematic meta-review
Source: Environ Health. 2023 Jan 5;22:2. doi: 10.1186/s12940-022-00955-8 (PMC9814186; doi:10.1186/s12940-022-00955-8)
Supplement: Supplementary file 1 — Additional file 1: Table S1. Explanation, measurement method and potential data source for neighborhood risk factors. Table S2. The search keywords for each neighborhood risk factor. Table S3. The checklist of AMSTAR. Table S4. Quality assessment results of all the included reviews. Table S5. Characteristics of all the included reviews. Table S6. Summary of reviews that compare findings for different population subgroups. [file 12940_2022_955_MOESM1_ESM.docx]

**Additional file 1: Appendix**

**Additional file 1: Table S1**. Explanation, measurement method and potential data source for neighborhood risk factors

| Risk factors | Explanation and measurement method  (The neighborhood refers to 300 / 500 / 1000 - m buffer surrounding the housing or the administrative area where the housing locates) | Data source |
| --- | --- | --- |
| 1. Green and blue spaces | 1. The green and blue space proportions in the neighborhood (m^2^/km^2^);  2. The tree canopy proportions in the neighborhood (m^2^/km^2^);  3. Tree proportions in human’s eyes in the neighborhood (measured through street view image by using machine learning tools, such as SegNet) (%);  4. Normalised difference vegetation index (NDVI) in the neighborhood (Dimensionless);  5. The presence of green / blue spaces (≥5000 m^2^) in the neighborhood (Y/N);  6. The Euclidean / road distance to the nearest green / blue space / park (≥5000 m^2^) (if green and blue spaces do not exist in every neighborhood) | Street view image / remote sensing image/ land use data |
| 2. Facilities for physical and leisure activities / playability of urban space | Relevant facilities: gym, playground (especially for children), stadium, swimming pool, facilities for physical exercises alongside the street.  1.The presence / density of facilities and open space in the neighborhood (Y/N; facilities/ km^2^);  2.The facility richness in the neighborhood (facility types per km^2^)  The facilities into 8 categories were divided to measure the mixing degree. This indicator was measured by the formula  a. *richness_facility* = -∑ (*p*_i_*ln *p*_i_), (*i*=1, 2, …, 8);  b. *p*_i_= *N*_i_/∑ (*N*_i_), (*i*=1, 2, …, 8);  c. *N*_i_=*B*_i_/*C*_i_, (*i*=1,2, …, 8).  where *B*_i_ represented the number of a specific facility type in the neighborhood, and *C*_i_ represented the total number of this facility type in the urban metropolitan area (Long & Liu, 2016).  3.The Euclidean / road distance to the nearest facility or open space (m) (if relevant facilities do not exist in every neighborhood). | Street view image / High spatial resolution remote sensing image |
| 3. Building density:  plot ratio (for 2D) /  floor area ratio (for 3D) | 1. For 2D, plot area, refers to the base area of buildings, divided by the neighborhood area (m^2^/km^2^);  2. For 3D, floor area ratio, refers to the total floor area of the buildings, divided by the neighborhood area (m^2^/km^2^). | High spatial resolution remote sensing image / building data / Google map |
| 4. Neighborhood disorder | The deterioration of landscape or the overall disorder of appearance of neighborhood, such as building façade damage / fouling / graffiti, unapproved construction, abandoned buildings, broken public space, unhardened road, abandoned cars, illegal street stalls, and so on. The total “disorder score” of each street view point can be obtained through 19 secondary categories for spatial disorder. | Street view image |
| 5. Walkability  (Medium)  * Walkability focuses on the quality of walkable streets created by various facilities along the streets, instead of focusing on road safety | A walkability index in the neighborhood  * The walkability index can incorporate part of indicators below: land use mix, street connectivity, net residential density, retail ﬂoor area ratios, population density, number of accessible destinations (banks, grocery stores, restaurants, etc.) and so on. | Street view image / land use data / census data / road network data / walk score |
| 6. Bikeability  * Also related to safety & injuries | 1.The presence / quality and materials of sidewalks and bike paths (Y/N; High/Medium/Low; Cement/Plastic cement/ etc.);  2.The sidewalks / bike paths proportions in the neighborhood (m/km^2^) | Street view image |
| 7. Accessibility to community-level health facilities | Relevant facilities:  community health center, pharmacy, clinic, AED equipment INSTEAD OF tertiary hospitals  1.The presence / density of relevant facilities in the neighborhood (Y/N; numbers / km^2^)  2.The Euclidean / road distance to the nearest relevant facility (if relevant facilities do not exist in every neighborhood) (m; Y/N) | POI data / Road network data |
| 8. Accessibility to infrastructure providing unhealthy food | Relevant facilities:  fast-food restaurants, dessert shops, and snack bars  The density of relevant facilities in the neighborhood (numbers / km^2^) | POI data / street view image |
| 9. Accessibility to fruit / vegetable shops and markets | 1. The presence / density of fruit / vegetable shops and markets in the neighborhood (none/≥1; numbers / km^2^)  2.The Euclidean / road distance to the nearest fruit / vegetable shop and market (if relevant facilities do not exist in every neighborhood) (m) | POI data / street view image |
| 10. Accessibility to bus / subway / metro stops | 1.The presence / density of bus / subway / metro stops in the neighborhood (Y/N; numbers / km^2^);  2.The Euclidean / road distance to the nearest bus / subway / metro stop (if relevant facilities do not exist in every neighborhood) (m; Y/N) | POI data / Google map |
| 11. Air pollution | Concentration of PM_2.5_, PM_10_, NO_x_, O_3_ in the neighborhood (μg/m^3^) | Air quality data / high spatial resolution remote sensing image |
| 12. Noise pollution | Noise levels (24 hours and night) in the neighborhood (dB) | Questionnaire / Noise mapping |
| 13. Soil pollution | The presence / concentration of soil pollution in the neighborhood (Y/N; mg/kg pollutants in soil) | Municipal management data  (Soil pollution data usually responsible by the urban environmental protection department) |
| 14. Level of neighborhood sanitation | The presence of waste disposal services / solid waste / trash piles / open gutters / open sewers / other liquid wastes in the neighborhood (Y/N) | Questionnaire / Street view image |
| 15. Surface water pollution | The presence of surface water pollution (Landscape water, rivers and lakes) in the neighborhood (recognized by eyes or monitoring data) (Y/N) | Street view data / Monitoring data |
| 16. Proximity to major roads / railways / subway lines / airports | The Euclidean / road distance to major roads/ railways / subway lines / airports, or the presence of facilities in the neighborhood (km; Y/N) | Road network data / POI data |
| 17. Proximity to industrial sites and brownfield sites | The Euclidean / road distance to industrial sites and brownfield sites, or the presence of sites above in the neighborhood (km; Y/N) | Land use data / POI data |
| 18. Proximity to landfills / garbage treatment plants | The presence of landfills / garbage treatment plants in the neighborhood, or The Euclidean / road distance to the nearest site above (Y/N; km) | Municipal management data/medium spatial resolution remote sensing image / Street view image/ POI data |
| 19. Vulnerability to floods | The Euclidean / road distance to floods, or the presence of floods in the neighborhood (km; Y/N) | Municipal management data |
| 20. Social structure of population  ——Residential segregation vs integration | The presence of segregation from the aspect of Ethnicity/race, income level (income distribution and poverty prevalence), employment rate, educational level, etc. in the neighborhood (Y/N; High/ Medium/ Low; %; PhD / Master / Bachelor / High school / Middle school / etc.) | Census data |
| 21. Population density | The total population number divided by the neighborhood area (population/km^2^) | Census data |
| 22. Accessibility to tobacco/ alcohol retailers and advertisements | The presence / density of tobacco / alcohol retailers and ads in the neighborhood (Y/N; numbers / km^2^) | POI data / street view image |
| 23. Perceived social trust / cohesion | The presence of community-based organizations [Group activities, the proprietors' committee, regulations of neighborhood, etc.] (Y/N);  The vacant / turnover rates of housing in the neighborhood (The number of vacant / turnover housings/ the whole housing number) | Social economic data/ Questionnaire |
| 24. Incidence of bullying, crimes and violence | The incidence of bullying, crimes and violence in the neighborhood (incidence /km^2^) | Questionnaire / Street view image / Police record |
| 25. Unfenced / unprotected dangerous sites nearby leading to falls | The presence of unfenced / unprotected railway, construction sites, drainageway, missing manhole covers, roofs, or slippery/broken ground surfaces nearby the neighborhood, puddles on the road in the neighborhood (Y/N) | Street view image / Questionnaire |
| 26. Unfenced / unprotected dangerous sites nearby leading to drowning | The presence of unfenced / unprotected water bodies nearby the neighborhood, including swimming pools, ponds and lakes in the neighborhood (Y/N) | Street view image / Questionnaire |
| 27. Unfenced / unprotected dangerous sites nearby leading to electrical burning | The presence of the tangled mess of overhead power cables, the visible/exposed wires on the ground/wall, electromagnetic radiation protection zone, radio-TV transmission facilities, electric station zone in the neighborhood (Y/N) | Street view image / Questionnaire |
| 28. Street animal bites | The presence of street animals in the neighborhood, such as dogs, snakes, and scorpions (Y/N) | Street view image / Questionnaire |
| 29. Insufficient street lighting leading to injury | 1.The coverage percentage of street lighting in the neighborhood (m^2^/km^2^);  2.The number of street lights per length of road in the neighborhood (light number / km) | Nightlight data/ Street view image |
| 30. Poor crossroads design threatening traffic safety | The presence of traffic lights, crosswalks, roundabouts, overpass / underpass, median refuge island, etc. in the neighborhood (Y/N) | Street view image/ high spatial resolution remote sensing image |
| 31. Intense traffic threatening traffic safety | The number and average speed of each type of vehicle (bus/ truck/coach/car) on the road per day in the neighborhood (vehicle number / speed) | Origin-Destination survey data/ Google map |
| 32. Insufficient traffic management threatening traffic safety | The presence of traffic management (including timing of traffic signal, signs, speed limit, traffic volume, limitation of trucks, etc.) in the neighborhood (Y/N) | Street view image/ high spatial resolution remote sensing image/ Questionnaire |
| 33. Insufficient pedestrian / cyclist-vehicle separation threatening traffic safety | The presence of pedestrian / cycling-vehicle separation design in the neighborhood (Y/N) | Street view image/ high spatial resolution remote sensing image |

**Additional file 1: Table S2**. The search keywords for each neighborhood risk factor

| Risk factor | KEYWORDS |
| --- | --- |
| Overall/general | “built environment” |
| Green and blue spaces | “green space” OR “blue space” |
| Facilities for physical and leisure activities | “physical activity facility” OR “exercise equipment” OR “recreational facility” OR “leisure facility” OR “playability” |
| Building density | “building density” |
| Neighborhood disorder | “physical disorder” OR “social disorder” OR “perceived disorder” |
| Walkability | “walkability” |
| Bikeability | “bikeability” OR “sidewalk” OR “bike path” |
| Accessibility to community-level health facilities | (“community health center” OR “community health facilities” OR “pharmacy” OR “clinic”) AND (“distance” OR “presence” OR “density” OR “access”) |
| Accessibility to infrastructure providing unhealthy food | (“fast food” OR “dessert” OR “snack” OR “retail food”) AND (“shop” OR “restaurant” OR “environment” OR “access” OR “bar”) |
| Accessibility to fruit/vegetable shops and markets | (“fruit” OR “vegetable”) AND (“shop” OR “market” OR “access”) |
| Accessibility to bus / subway / metro stops | “bus” OR “subway” OR “metro” |
| Soil pollution | “soil pollution” |
| Level of neighborhood sanitation | “neighborhood sanitation” |
| Surface water pollution | “water pollution” |
| Proximity to major roads / railways / subway lines / airports | (“road” OR “roadway” OR “subway” OR “airport” OR “railway”) AND (“distance” OR “proximity” OR “density”) |
| Proximity to industry | (“industry” OR “industrial” OR “brownfield” OR “polluted sites”) AND (“distance” OR “proximity” OR “density”) |
| Proximity to landfills / garbage treatment plants | (“landfill” OR “waste” OR “garbage”) AND (“distance” OR “proximity” OR “density”) |
| Accessibility to infrastructure providing tobacco and alcohol | (“tobacco” OR “alcohol” OR “wine” OR “liquor”) AND (“shop” OR “retailer”) |

**Additional file 1: Table S3**. The checklist of AMSTAR

| Questions | Answers |
| --- | --- |
| 1. Was an ‘a priori’ design provided?  The research question and inclusion criteria should be established before the conduct of the review. | Yes  No  Can’t answer  Not applicable |
| 2. Was there duplicate study selection and data extraction?  There should be at least two independent data extractors and a consensus procedure for disagreements should be in place. | Yes  No  Can’t answer  Not applicable |
| 3. Was a comprehensive literature search performed?  At least two electronic sources should be searched. The report must include years and databases used (e.g. Central, EMBASE, and MEDLINE). Key words and/or MESH terms must be stated and where feasible the search strategy should be provided. All searches should be supplemented by consulting current contents, reviews, textbooks, specialized registers, or experts in the particular field of study, and by reviewing the references in the studies found. | Yes  No  Can’t answer  Not applicable |
| 4. Was the status of publication (i.e. grey literature) used as an inclusion criterion?  The authors should state that they searched for reports regardless of their publication type. The authors should state whether or not they excluded any reports (from the systematic review), based on their publication status, language etc. | Yes  No  Can’t answer  Not applicable |
| 5. Was a list of studies (included and excluded) provided?  A list of included and excluded studies should be provided. | Yes  No  Can’t answer  Not applicable |
| 6. Were the characteristics of the included studies provided?  In an aggregated form such as a table, data from the original studies should be provided on the participants, interventions and outcomes. The ranges of characteristics in all the studies analyzed e.g. age, race, sex, relevant socioeconomic data, disease status, duration, severity, or other diseases should be reported. | Yes  No  Can’t answer  Not applicable |
| 7. Was the scientific quality of the included studies assessed and documented?  ‘A priori’ methods of assessment should be provided (e.g., for effectiveness studies if the author(s) chose to include only randomized, double-blind, placebo controlled studies, or allocation concealment as inclusion criteria); for other types of studies alternative items will be relevant. | Yes  No  Can’t answer  Not applicable |
| 8. Was the scientific quality of the included studies used appropriately in formulating conclusions?  The results of the methodological rigor and scientific quality should be considered in the analysis and the conclusions of the review, and explicitly stated in formulating recommendations. | Yes  No  Can’t answer  Not applicable |
| 9. Were the methods used to combine the findings of studies appropriate?  For the pooled results, a test should be done to ensure the studies were combinable, to assess their homogeneity (i.e. Chi-squared test for homogeneity, I²). If heterogeneity exists a random effects model should be used and/or the clinical appropriateness of combining should be taken into consideration (i.e. is it sensible to combine?). | Yes  No  Can’t answer  Not applicable |
| 10. Was the likelihood of publication bias assessed?  An assessment of publication bias should include a combination of graphical aids (e.g., funnel plot, other available tests) and/or statistical tests (e.g., Egger regression test). | Yes  No  Can’t answer  Not applicable |
| 11. Was the conflict of interest stated?  Potential sources of support should be clearly acknowledged in both the systematic review and the included studies. | Yes  No  Can’t answer  Not applicable |

**Additional file 1: Table S4.** Quality assessment results of all the included reviews

| Reviews | Question 1 | Question 2 | Question 3 | Question 4 | Question 5 | Question 6 | Question 7 | Question 8 | Question 9 | Question 10 | Question 11 | Total score |
| --- | --- | --- | --- | --- | --- | --- | --- | --- | --- | --- | --- | --- |
| **Boonhat and Lin., 2020** | **1** | **1** | **1** | **0** | **1** | **1** | **1** | **1** | **1** | **1** | **1** | **10** |
| **Jephcote et al., 2020** | **1** | **1** | **1** | **0** | **1** | **1** | **1** | **1** | **1** | **1** | **1** | **10** |
| **Lin et al., 2017** | **1** | **1** | **1** | **0** | **0** | **1** | **1** | **1** | **1** | **1** | **1** | **9** |
| **Yuan et al, 2020** | **1** | **1** | **1** | **0** | **0** | **1** | **1** | **1** | **1** | **1** | **1** | **9** |
| **Twohig-Bennett et al, 2018** | **1** | **1** | **1** | **0** | **0** | **1** | **1** | **1** | **1** | **1** | **1** | **9** |
| **Filippini et al., 2019** | **1** | **1** | **1** | **0** | **0** | **1** | **1** | **1** | **1** | **1** | **1** | **9** |
| **Den Braver et al., 2018** | **1** | **1** | **1** | **0** | **0** | **1** | **1** | **1** | **1** | **1** | **1** | **9** |
| **Gascon et al, 2016** | **1** | **1** | **1** | **0** | **0** | **1** | **1** | **0** | **1** | **1** | **1** | **8** |
| **Di et al., 2020** | **1** | **1** | **1** | **0** | **0** | **1** | **1** | **0** | **1** | **1** | **1** | **8** |
| **Chandrabose et al., 2019** | **1** | **1** | **1** | **0** | **0** | **1** | **1** | **1** | **1** | **0** | **1** | **8** |
| **Lin et al., 2018** | **1** | **1** | **1** | **0** | **0** | **1** | **1** | **1** | **1** | **1** | **0** | **8** |
| **Boothe et al., 2014** | **1** | **1** | **1** | **1** | **0** | **1** | **1** | **0** | **1** | **1** | **0** | **8** |
| **Rugel et al, 2020** | **1** | **1** | **1** | **0** | **0** | **1** | **1** | **1** | **0** | **0** | **1** | **7** |
| **Rigolon et al, 2021** | **1** | **1** | **1** | **0** | **0** | **1** | **1** | **1** | **0** | **0** | **1** | **7** |
| **Raffetti et al., 2019** | **1** | **1** | **1** | **0** | **0** | **1** | **1** | **1** | **0** | **0** | **1** | **7** |
| **Malambo et al., 2016** | **1** | **1** | **1** | **0** | **1** | **1** | **1** | **0** | **0** | **0** | **1** | **7** |
| **Kondo et al, 2018** | **1** | **1** | **1** | **0** | **0** | **1** | **1** | **1** | **0** | **0** | **1** | **7** |
| **Jilani et al., 2020** | **1** | **1** | **1** | **0** | **0** | **1** | **1** | **1** | **0** | **0** | **1** | **7** |
| **Fazzo et al., 2017** | **1** | **1** | **1** | **0** | **0** | **1** | **1** | **1** | **0** | **0** | **1** | **7** |
| **De la fuente et al, 2021** | **1** | **1** | **1** | **0** | **0** | **1** | **1** | **1** | **0** | **0** | **1** | **7** |
| Gasana et al., 2012 | 1 | 1 | 1 | 0 | 0 | 1 | 1 | 1 | 0 | 0 | 1 | 7 |
| Zhao et al., 2017 | 1 | 0 | 1 | 0 | 0 | 1 | 1 | 1 | 1 | 1 | 0 | 7 |
| Filippini et al., 2015 | 1 | 0 | 1 | 0 | 0 | 1 | 1 | 1 | 1 | 1 | 0 | 7 |
| Salgado et al, 2020 | 1 | 1 | 1 | 0 | 0 | 1 | 1 | 0 | 0 | 0 | 1 | 6 |
| Hamra et al., 2015 | 1 | 0 | 0 | 0 | 1 | 1 | 0 | 0 | 1 | 1 | 1 | 6 |
| Dendup et al, 2018 | 1 | 1 | 1 | 0 | 0 | 1 | 1 | 0 | 0 | 0 | 1 | 6 |
| Peters et al., 2019 | 1 | 1 | 1 | 0 | 0 | 1 | 1 | 1 | 0 | 0 | 0 | 6 |
| Gascon et al, 2017 | 1 | 1 | 1 | 0 | 0 | 1 | 1 | 1 | 0 | 0 | 0 | 6 |
| Dzhambov et al., 2016 | 1 | 1 | 1 | 0 | 0 | 1 | 1 | 1 | 0 | 0 | 0 | 6 |
| Delgado-Saborit et al., 2021 | 1 | 0 | 1 | 1 | 0 | 1 | 0 | 0 | 0 | 0 | 1 | 5 |
| Browning et al, 2017 | 1 | 0 | 0 | 0 | 0 | 1 | 1 | 1 | 0 | 0 | 1 | 5 |
| Vinti et al., 2021 | 1 | 0 | 1 | 0 | 0 | 1 | 1 | 1 | 0 | 0 | 0 | 5 |
| Kraft et al., 2020 | 1 | 0 | 1 | 0 | 0 | 1 | 1 | 1 | 0 | 0 | 0 | 5 |
| Schulz et al, 2018 | 1 | 0 | 1 | 0 | 0 | 1 | 0 | 0 | 0 | 0 | 1 | 4 |
| Hartley et al, 2020 | 1 | 0 | 1 | 0 | 0 | 1 | 1 | 0 | 0 | 0 | 0 | 4 |
| Buteau et al., 2019 | 1 | 1 | 0 | 0 | 0 | 1 | 0 | 0 | 1 | 0 | 0 | 4 |
| Lai et al, 2019 | 1 | 0 | 0 | 0 | 1 | 1 | 0 | 0 | 0 | 0 | 0 | 3 |
| Kabisch et al, 2017 | 1 | 0 | 1 | 0 | 0 | 1 | 0 | 0 | 0 | 0 | 0 | 3 |
| Rojas-Rueda et al, 2021 | 1 | 1 | 0 | 0 | 0 | 1 | 0 | 0 | 0 | 0 | 0 | 3 |
| Gowers et al., 2012 | 1 | 0 | 0 | 0 | 0 | 0 | 0 | 0 | 0 | 0 | 0 | 1 |

**Additional file 1: Table S5.** Characteristics of all the included reviews

|  | **Reviews** | **Participants** | **Outcomes** | **Risk factor examined** | **No. of studies included** | **Sample range** | **Primary study type** | **Type of review** | **Quality assessment** | **Evidence strength** |
| --- | --- | --- | --- | --- | --- | --- | --- | --- | --- | --- |
| Accessibility to infrastructure providing unhealthy food | Malambo et al., 2016 | Adults | Stroke and CVDs | Density of fast food restaurants | 18 (2) | n=2411, n=4,319,674 | Longitudinal (1), cross-sectional (1) | Systematic review | high | medium |
|  | Kraft et al., 2020 | US Low-SES general population | Stroke | Food access | 43 (1) | n=1,247 | Cross-sectional (1) | Systematic review | moderate | limited |
|  | Den Braver et al., 2018 | General population | T2DM | Food access | 86(20) | n=832, n=2,948,851 | Longitudinal (7), cross-sectional (13) | Meta-analysis | high | strong |
|  | Dendup et al, 2018 | Adults, 18-94 years old | Diabetes | Healthy food environment | 16 | n=46, n=4,718,583 | Cohort study (6) Ecological (6) Cross-sectional (4) | Systematic review | moderate | strong |
| Proximity to major road | Boothe et al., 2014 | Children | Childhood leukemia | Residential traffic exposure | 9 | n=98/262, n=1,728/3,456 | Case-control (8) Population-based study (1) | Meta-analysis | high | strong |
|  | Filippini et al., 2019 | Children | Childhood leukemia | Residential traffic exposure, residential proximity to repair garages or petrol stations | 26 (20 traffic) | n= 128/128, n=532 (416)/2,096,402 (traffic) | Case-control (19 traffic), cohort (1 traffic) | Meta-analysis | high | strong |
|  | Filippini et al., 2015 | Children | Childhood leukemia | Residential traffic exposure | 26 (14 traffic) | n=130/251, n=1,989/5,506 (traffic) | Case-control (13 traffic) Ecological study (1 traffic) | Meta-analysis | Moderate | strong |
|  | Filippini et al., 2015 | Children | Childhood leukemia | Residential proximity to repair garages or petrol stations | 26 (4 petrol or garages) | n=280/285, n=1928/3456 | Case-control (4 petrol) | Meta-analysis | Moderate | medium |
|  | Delgado-Saborit et al., 2021 | Adults | Dementia | Residential traffic exposure | 69 | n=200, n=350844 (traffic) | Cross-sectional (1 traffic) | Systematic review | Moderate | limited |
|  | Peters et al., 2019 | Adults | Dementia | Residential proximity to major roads | 13 (2 residential proximity to major roads) | n=130978, n=243611 (residential proximity to major roads) | Cohort study (2 residential proximity to major roads) | Systematic review | Moderate | medium |
|  | Di et al., 2020 | Adults | Rheumatoid arthritis (RA) | Residential traffic exposure | 8(2 traffic) | n=121700, n=640041 (traffic) | Prospective cohort study (1traffic) Nested case-control study (1 traffic) | Meta-analysis | high | medium |
|  | Dzhambov et al., 2016 | Adults | Rheumatoid Arthritis | Residential proximity to major roads | 6(2 traffic) | n=121700, n=640041 (traffic) | Prospective cohort study (1 traffic) Nested case-control study (1 traffic) | Meta-analysis | moderate | medium |
|  | Gasana et al., 2012 | Children | Children Asthma | Residential proximity to major roads | 19 (1 traffic) | n=6,683 | Cross-sectional (1 traffic) | Meta-analysis | moderate | limited |
|  | Salgado et al, 2020 | Adults and children | Asthma | Traffic density | 1 | n=33,632 | Cross-sectional (1 traffic) | Systematic review | moderate | limited |
|  | Hamra et al., 2015 | General population | Lung cancer | Distance to roadways or traffic volume | 20(7 traffic) | n=1,648/97,865, n=12208/1265058 (traffic) | Cohort study (7 traffic) | Meta-analysis | moderate | strong |
|  | Jilani et al., 2020 | Adults | CVDs | Residential proximity to major roads | 18 (10 residential proximity to major roads) | n=509, n=8,168 (residential proximity to major roads) | Cross-sectional (8 traffic) cohort (2 traffic) | Systematic review | high | medium |
|  | Malambo et al., 2016 | 25+ | CVDs | Residential proximity major roads | 18 (1) | n=2,411 | Cross-sectional (1) | Systematic review | high | limited |
|  | Salgado et al, 2020 | Adults | CVD mortality | Road density | 1 | n=9,805 | Case-crossover (1) | Systematic review | moderate | limited |
|  | Malambo et al., 2016 | 45-64 | CHD | Traffic density | 18 (1) | m=13,309 | Survey (1) | Systematic review | high | limited |
|  | Zhao et al., 2017 | Adults | T2DM | Residential proximity to major roads | 8 | n=513, n=74,412 | Cohort study (6) Cross-sectional (2) | Meta-analysis | moderate | strong |
|  | Dendup et al, 2018 | Adults | Diabetes | Distance to roadways | 7 | n=2,124, n=89,460 | Cohort study (1) Cross-sectional (5) | Systematic review | moderate | medium |
|  | Salgado et al, 2020 | Adults | Diabetes | Traffic intensity | 1 | n=513 | Cross-sectional (1) | Systematic review | moderate | limited |
| Proximity to industry | Boonhat and Lin., 2020 | General population | Leukemia incidence and mortality | Residential exposure to petrochemical industry complexes (PICs) | 13 | n=2, n=92,071 | Cohort (9) Case-control (3) | Meta-analysis | very high | strong |
|  | Jephcote et al., 2020 | General population | Leukemia | Residential exposure to petrochemical industry complexes (PICs) | 13 | n=2, n=92,071 | Cohort (9) Case-control (3) | Meta-analysis | very high | strong |
|  | Jephcote et al., 2020 | General population | Non-Hodgkin’s Lymphoma | Residential exposure to petrochemical industry complexes (PICs) | 6 | n=92, n=54,000 | Cohort (7)  Case-control (2) | Meta-analysis | very high | strong |
|  | Jephcote et al., 2020 | General population | Hodgkin’s Lymphoma | Residential exposure to petrochemical industry complexes (PICs) | 9 | n=138, n=66,563 | Cohort (6) | Meta-analysis | very high | strong |
|  | Jephcote et al., 2020 | General population | Multiple Myeloma | Residential exposure to petrochemical industry complexes (PICs) | 3 | n=138, n=54,000 | Cohort (3) | Meta-analysis | very high | strong |
|  | Lin et al., 2017 | General population | Lung cancer | Residential exposure to petrochemical industry complexes (PICs) | 7 | n=95, n= 977,853 | Cohort (6) Case-control (1) | Meta-analysis | high | strong |
|  | Lin et al., 2018 | General population | Lung cancer | Residential exposure to petrochemical industry complexes (PICs) | 6 | n=437, n=396,517 | Cohort study (6) | Meta-analysis | high | strong |
|  | Raffetti et al., 2019 | General population | Respiratory tract diseases | Residential exposure to plants | 24 (8 respiratory tract diseases) | n=88, n=general population | Cross-sectional (5) case-control (1) Ecologic (1) Time series analysis (1) | Systematic review | high | medium |
|  | Raffetti et al., 2019 | General population | CVDs | Residential exposure to plants | 24 (3 CVD) | n=88, n=6,248 | Cross-sectional (1) Ecology (1) Case-control (1) | Systematic review | high | medium |
|  | Fazzo et al., 2017 | General population | Asthma | Residential living near hazardous waste sites | 58 (4 asthma) | Not available from the review | Ecological (1) Descriptive (2) Cohort (1) | Systematic review | high | medium |
|  | Fazzo et al., 2017 | General population | Liver cancer | Residential living near hazardous waste sites | 57 (7 liver cancer) | Not available from the review | Ecological (6) Descriptive (1) Meta-analysis (1) Cohort (1) | Systematic review | high | strong |
|  | Fazzo et al., 2017 | General population | Breast cancer | Residential living near hazardous waste sites | 57 (5 breast cancer) | Not available from the review | Ecological (4) Cohort (1) | Systematic review | high | medium |
|  | Fazzo et al., 2017 | General population | Bladder cancer | Residential living near hazardous waste sites | 57 (10 bladder cancer) | Not available from the review | Ecological (7) Descriptive (1) Meta-analysis (1) Cohort (1) | Systematic review | high | strong |
|  | Fazzo et al., 2017 | General population | Non-Hodgkin Lymphoma | Residential living near hazardous waste sites | 57 (9 Non-Hodgkin Lymphoma) | Not available from the review | Ecological (8) Cohort (1) | Systematic review | high | strong |
| Proximity to landfills | Vinti et al., 2021 | General population | Respiratory diseases | Residential exposure to municipal solid waste (MSW) | 29(6) | n=343, n=242409 | Cross-sectional (4) Cohort (2) | Systematic review | moderate | medium |
|  | Vinti et al., 2021 | General population | Cardiovascular diseases | Residential exposure to municipal solid waste (MSW) | 29(2) | n=, n=242409 | Cohort (1) Case-control (1) | Systematic review | moderate | medium |
| Walkability | Chandrabose et al., 2019 | Adults | T2DM outcomes | Walkability | 36(6) | n=583, n=1239262 | Cohort (3) | Meta-analysis | high | strong |
|  | Den Braver et al., 2018 | General population | T2DM outcomes | Walkability | 86(11) | n=583, n= 4,505,000 | Longitudinal (4), cross-sectional (2) | Meta-analysis | high | strong |
|  | Malambo et al., 2016 | Adults | T2DM outcomes | Walkability | 18 (2) | n=5970, n=512061 | Survey (1), cross-sectional (1) | Systematic review | high | weak |
|  | Dendup et al, 2018 | Adults | Diabetes | Walkability | 7 | n=3205, n=2770000 | Cohort (4) Ecological (1) Cross-sectional (2) | Systematic review | moderate | strong |
|  | Chandrabose et al., 2019 | Mid-old | CHD death | Land use mix | 36(1) | n=45376 | Observational study | Meta-analysis | high | limited |
|  | Chandrabose et al., 2019 | Mid-old | CHD | Street connectivity | 36(1) | n=45376 | Observational study | Meta-analysis | high | limited |
| Facilities for physical activity or recreation | Chandrabose et al., 2019 | Mid-old | Coronary heart disease (CHD) and Stroke | Recreational facilities | 36(3) | n=2,165,000 , n=4,194,252 | Cohort (3) | Meta-analysis | high | strong |
|  | Chandrabose et al., 2019 | Adults | Diabetes outcomes | Recreational facilities | 36(3) | n=2285, n=5124 | Cohort (2) | Meta-analysis | high | medium |
|  | Den Braver et al., 2018 | General population | T2DM outcomes | Facilities for physical activity | 86(6) | n=2157, n=3661 | Longitudinal (3), cross-sectional (3) | Meta-analysis | high | strong |
|  | Dendup et al, 2018 | Adults, 15-94 years old | Diabetes | Physical activity resources | 7 | n=2026, n=5124 | Cohort (3) Ecological (2) Cross-sectional (2) | Systematic review | moderate | strong |
| Green space | Chandrabose et al., 2019 |  | Total CVD | Green space | 36(1) | n=5112 | Cohort (1) | Meta-analysis | high | limited |
|  | Gascon et al, 2016 | Adults | Mortality of CVD | Greenness (percentage of green space in an area or NDVI) | 7 | n=5112, n=28600000 | Cohort (2) Ecological (4) Cross-sectional (1) | Meta-analysis | high | strong |
|  | Kondo et al, 2018 | General population | CVD mortality (including IHD/CHD, stroke) | Urban green space | 4 | n=1645, n=1170343 | Cohort (4) | Systematic review | high | strong |
|  | Rigolon et al, 2021 |  | CVD | Urban green space | 15 | n=408, n=116000000 | Cohort (6) Case-control (1) Ecological (2) Cross-sectional (6) | Systematic review | high | strong |
|  | Yuan et al, 2020 | Elders, 60~93 years old | CVD mortality | Urban green space | 4 | n=3544, n=162189 | Cohort study (4) | Meta-analysis | high | strong |
|  | Yuan et al, 2020 | Elders, 60~93 years old | CVD morbidity | Urban green space | 13 | n=912, n=5988606 | Cohort (8) Cross-sectional (5) | Meta-analysis | high | strong |
|  | Twohig-Bennett et al, 2018 | Adults | Cardiovascular mortality | Green space | 2 | n=250793, n=3749150 | Cohort (1) Cross-sectional (1) | Meta-analysis | high | limited |
|  | Browning et al, 2017 | General population | CVD | Greenness | 3 | n=, n=345143 | Cohort (1) Cross-sectional (2) | Systematic review | moderate | medium |
|  | Yuan et al, 2020 | Elders, 60~93 years old | IHD mortality | Urban green space | 3 | n=3544, n=108630 | Cohort study (3) | Meta-analysis | high | strong |
|  | Twohig-Bennett et al, 2018 | Adults | Coronary heart disease | Green space | 2 | n=5112, n=250793 | Cohort (2) | Meta-analysis | high | medium |
|  | Yuan et al, 2020 | Elders, 60~93 years old | Stroke mortality | Urban green space | 4 | n=3544, n=108630 | Cohort study (4) | Meta-analysis | high | strong |
|  | Twohig-Bennett et al, 2018 | Adults | Stroke | Green space | 3 | n=822, n=250793 | Cohort (3) | Meta-analysis | high | strong |
|  | Den Braver et al., 2018 | General population | T2DM outcomes | Green space | 86(7) | n=832, n=3,920,000 | Longitudinal (2), cross-sectional (5) | Meta-analysis | high | medium |
|  | De la fuente et al, 2021 | Adults, 15-85 years old | Diabetes | Green space | 7 | n=3751, n=345143 | Cohort (3) Cross-sectional (4) | Systematic review | high | strong |
|  | Kondo et al, 2018 | General population | Diabetes | Urban green space | 2 | n=3205, n=108603 | Cohort (2) | Systematic review | high | medium |
|  | Rigolon et al, 2021 |  | Diabetes | Urban green space | 7 | n=15477, n=116000000 | Cohort (1) Ecological (1) Cross-sectional (5) | Systematic review | high | medium |
|  | Twohig-Bennett et al, 2018 | Adults | Type II diabetes | Green space | 6 | n=822, n=250793 | Cohort (4) Cross-sectional (2) | Meta-analysis | high | strong |
|  | Browning et al, 2017 | General population | Diabetes | Greenness | 2 | n=4796, n=345143 | Cohort (1) Cross-sectional (1) | Systematic review | moderate | medium |
|  | Dendup et al, 2018 | General population | Diabetes | Green space/tree canopy/open space | 6 | n=2746, n=343103 | Cohort (1) Cross-sectional (5) | Systematic review | moderate | medium |
|  | Gascon et al, 2017 | Adults | Diabetes | Blue space (coastal VS continental) | 2 | n=3054, n=10242 | Cross-sectional (2) | Systematic review | moderate | weak |
|  | Kondo et al, 2018 | General population | Respiratory disease mortality | Urban green space | 3 | n=108603, n=1170343 | Cohort (3) | Systematic review | high | strong |
|  | Yuan et al, 2020 | Elders, 60~93 years old | Respiratory mortality | Urban green space | 5 | n=3544, n=162189 | Cohort study (5) | Meta-analysis | high | strong |
|  | Rugel et al, 2020 | Adults | Respiratory diseases | TRAP-natural spaces | 6 | n=41688, n=660505 | Cohort (2) Case-control (1) Ecological (3) | Systematic review | high | strong |
|  | Twohig-Bennett et al, 2018 | Children | Asthma | Green space | 2 | n=1389, n=1489 | Case-control (1) Cross-sectional (1) | Meta-analysis | high | weak |
|  | Rigolon et al, 2021 |  | Atopic diseases (asthma, eczema) | Urban green space | 11 | n=1178, n=116000000 | Cohort (5) Ecological (1) Cross-sectional (5) | Systematic review | high | strong |
|  | Browning et al, 2017 | General population | Atopic diseases (asthma, eczema, rhinitis) | Greenness | 7 | n=150, n=345143 | Cohort (3) Cross-sectional (4) | Systematic review | moderate | strong |
|  | Rigolon et al, 2021 |  | Cancer | Urban green space | 1 | n=3927 | Case-control (1) | Systematic review | high | limited |
|  | Browning et al, 2017 | General population | Cancer | Greenness | 2 | n=3927, n=345143 | Cross-sectional (2) | Systematic review | moderate | weak |
|  | Gascon et al, 2016 | Adults | Mortality of lung cancer | Greenness (percentage of green space in an area or NDVI) | 3 | n=1546405, n=28600000 | Ecological (2) Cross-sectional (1) | Meta-analysis | high | weak |
|  | Kondo et al, 2018 | General population | Prostate cancer | Urban green space | 1 | n=3927, n=108603 | Case-control (1) | Systematic review | high | limited |
|  | Kondo et al, 2018 | General population | Kidney disease mortality | Urban green space | 1 | n=108603 | Cohort (1) | Systematic review | high | limited |

**Additional file 1: Table S6.** Summary of reviews that compare findings for different population subgroups

| AUTHOR/DATE* | Boonhat and Lin., 2020[54] | Boothe et al., 2014[10] | Rigolon et al, 2021[45] | Rigolon et al, 2021[45] | Yuan et al, 2020[46] | De la fuente et al, 2021[50] | Lin et al., 2018[56] |
| --- | --- | --- | --- | --- | --- | --- | --- |
| Neighborhood related risk factor | Proximity to industrials | Proximity to major road | Green space | Green space | Green space | Green space | Proximity to industrials |
| NCD outcome | Leukemia incidence and mortality | Childhood leukemia | Atopic diseases | CVD | CVD mortality | Diabetes | Lung cancer |
| Gender |  |  |  |  |  | Gender differences should be considered. | A greater harmful effect was found for females. |
| Age |  |  |  |  | A reduced incidence/prevalence of major CVD outcomes was found in older individuals. |  |  |
| Ethnicity |  |  | No significant difference |  |  |  |  |
| Socio-economic Status |  | No significant difference | A greater protective effect was found for low-SES people and neighborhoods. | A greater protective effect was found for low-SES people. |  |  |  |
| Geographical region or country |  | No significant difference |  | A greater protective effect was found for Europe groups than North America groups. |  |  | A greater harmful effect was found for Europe groups. |
| Follow-up period | A greater harmful effect was found for groups with follow-up periods of ≥10 years | No significant difference |  |  |  |  |  |
| Study period |  | No significant difference |  |  |  |  |  |
| Study quality score |  | No significant difference |  |  |  |  |  |

*****Only reviews that reported data on differential health effects (or associations) of neighborhood infrastructure related risk factors on population subgroups (defined by gender, age, ethnicity, and etc.) are featured in this table.
